# Reply to Commentary on Article: iCare Technique of Dissolving Ellanse M Nodules Using Collagenase: A Case Series and Experimental Study

**DOI:** 10.1111/jocd.70512

**Published:** 2025-10-29

**Authors:** Larry Wu

**Affiliations:** ^1^ iCare Medical Centre Singapore

**Keywords:** collagen, hyaluronidase, ultrasound

Ellanse, a polycaprolactone‐based collagen stimulator, has been associated with abnormal collagen formation such as nodules, which has an incidence of 0.023% [1]. The management of nodules includes triamcinolone injection, consumption of methotrexate tablets, and surgical excision [2]. In our article, iCare Technique of Dissolving Ellanse M Nodules Using Collagenase: A Case Series and Experimental Study [3], collagenase has been demonstrated to be effective in addressing nodule formation associated with Ellanse M.

Collagenase is defined as a therapeutic enzyme that degrades collagen, a major protein found in connective tissues [4]. It can be used to treat conditions involving abnormal collagen such as Dupuytren's contracture and keloids [5]. Collagenases are a broad class of enzymes distinguished by their diverse substrate specificities and varied origins. These include bacterial collagenases (e.g., *Clostridium histolyticum* collagenase), human matrix metalloproteinases (MMPs) like MMP‐1, MMP‐8, and MMP‐13, and recombinant collagenases [6]. The collagenase used in our article is a recombinant source of collagenase and is not Qwo distributed by Endo International. The inherent presence of adventitious agents or process‐related impurities within purified bacterial collagenase (Qwo) preparations can provoke allergic responses in recipients. As inferred from the correspondence's references [1, 4, 5, 6], all articles point towards collagenase derived from *Clostridium histolyticum* , which is not relevant to our article.

I would like to further draw your attention to another article, ‘**Point‐of‐Care Ultrasound (POCUS) for Precision Management in Ellanse‐Treated Patients**,’ published on 31 May 2025 in the *Journal of Cosmetic Dermatology*, written by Wu et al. [7] The left chin nodule in case 4 was treated with ultrasound‐directed injection of collagenase that is five times the estimated volume of the nodule. This illustrates a highly targeted, ultrasound‐guided intralesional quantified approach. Furthermore, in the article, POCUS has been useful in analyzing the vascular anatomy for Ellanse‐treated patients prior to further treatment due to the presence of neocollagenesis. In our patient with a chin nodule, the use of POCUS will allow the left submental artery to be imaged and ensure that the injection is directed away from the left submental artery, hence avoiding inadvertent intravascular injection. Despite the reported rarity of nodules associated with Ellanse (0.023%), the iCare method of Ellanse dissolution has been used in 23 nodules without significant complications and with satisfactory cosmesis. Data will be published once we accumulate a statistically significant amount for robust scientific review. The combination of POCUS‐directed delivery of collagenase with POCUS analysis of vascular anatomy has been the standard of care proposed by our article.

The ultrasound‐directed method starkly differs from cellulite treatment, as noted in the correspondence. Cellulite is characterized by fat deposits pushing through connective tissue beneath the skin, creating an uneven surface [8]. In addition, the skin is tethered by fibrous connective tissue bands (septa) to the underlying subcutaneous tissue. In cellulite, a more extensive injection targeting the fibroseptal across significantly larger areas, such as the upper arms, thighs, or buttocks, is recommended. This inherently demands a much greater volume of collagenase, thereby escalating the risk of complications like hematoma and allergy.

In summary, the series of 2 articles—(1) iCare Technique of Dissolving Ellanse M Nodules Using Collagenase: A Case Series [3] and Experimental Study and (2) Point‐of‐Care Ultrasound (POCUS) for Precision Management in Ellanse‐Treated Patients [7]—have been specially prepared to assist Ellanse practitioners in managing difficulties encountered during Ellanse treatment. The correspondence's analogy using Qwo collagenase (a collagenase of bacterial origin) and cellulite (different pathology) is unsubstantiated and insufficient to warrant any definitive inference. The use of the iCare method of dissolving Ellanse M nodules, combined with POCUS‐directed treatment, represents a significant advancement in the management of Ellanse‐treated patients. This targeted use of collagenase offers distinct advantages over methotrexate consumption (due to toxicity) and surgery (due to scarring).

## Conflicts of Interest

The author declares no conflicts of interest.

## Data Availability

The data that support the findings of this study are available from the corresponding author upon reasonable request.
